# Bioactive Phytochemicals from *Artocarpus integer* Leaves Improve Bone-Related Outcomes in Ovariectomized Rats: An Integrated LC–HRMS, Network Pharmacology, and Experimental Study

**DOI:** 10.3390/nu18172874

**Published:** 2026-09-02

**Authors:** Anton Bahtiar, Amelia Novia Angie, Tri Wahyuni, Sirithon Siriamornpun

**Affiliations:** 1 Disease-Related Biomarker and Prospective Therapy Cluster, Faculty of Pharmacy, Universitas Indonesia, Kampus UI, Depok 16424, Indonesiawahyuni.tri@farmasi.ui.ac.id (T.W.); 2 Research Unit of Thai Food Innovation, Department of Food Technology, Faculty of Technology, Mahasarakham University, Mahasarakham 44150, Thailand; sirithon.s@msu.ac.th

**Keywords:** *Artocarpus integer*, ovariectomized rat, network pharmacology, osteopontin, prenylated flavonoids, bone remodeling

## Abstract

Background: Osteoporosis is a multifactorial skeletal disorder characterized by reduced bone mass, impaired bone remodeling, and an increased risk of fractures, particularly under estrogen-deficient conditions. *Artocarpus integer* (Thunb.) Merr. contains prenylated flavonoids and chalcone derivatives with diverse biological activities; however, its anti-osteoporotic potential remains largely unexplored. This study investigated the phytochemical composition, molecular mechanisms, and anti-osteoporotic effects of *A. integer* leaf extract in an ovariectomized (OVX) rat model. Methods: Phytochemical profiling was performed using liquid chromatography–high-resolution mass spectrometry (LC–HRMS). Network pharmacology was employed to identify potential osteoporosis-related targets and signaling pathways. The anti-osteoporotic activity of the extract was evaluated in OVX rats through physiological and biochemical assessments, including body weight gain, uterine weight, serum biomarkers, femoral calcium content, and RT-PCR analysis of genes associated with osteogenesis, osteoclastogenesis, and estrogen signaling. Results: LC–HRMS analysis identified several bioactive compounds, including isobavachalcone, artocarpesin, morachalcone A, genistein, apigenin, luteolin, naringenin, catechin derivatives, and mangiferin. Network pharmacology revealed 96 overlapping targets between *A. integer* phytochemicals and osteoporosis-related genes, highlighting pathways involved in estrogen signaling, PI3K–Akt signaling, osteoclast differentiation, inflammation, and metabolic regulation. In vivo, OVX rats exhibited increased body weight gain, uterine atrophy, elevated leptin levels, reduced adiponectin concentrations, and decreased femoral calcium content. Treatment with *A. integer* attenuated OVX-induced metabolic alterations, improved adipokine profiles, and increased femoral calcium content, particularly in the medium-dose group. RT-PCR analysis demonstrated the upregulation of the osteogenic markers *Runx2* and *Osx*, together with the downregulation of the osteoclastogenic markers *TRAP*. Conclusions: *Artocarpus integer* leaf extract exhibited promising anti-osteoporotic activity through the coordinated regulation of osteogenesis, osteoclastogenesis, estrogen-related signaling, and bone mineral preservation. These findings support the potential development of *A. integer* as a nutraceutical candidate for the prevention or management of postmenopausal osteoporosis.

## 1. Introduction

Osteoporosis is a progressive skeletal disorder characterized by reduced bone mass and deterioration of bone microarchitecture, resulting in decreased bone strength and increased fracture risk [1,2]. Bone loss occurs due to an imbalance between bone formation and bone resorption, in which osteoclastic activity exceeds osteoblastic bone formation. Peak bone mass is generally achieved during the third decade of life, after which bone resorption progressively surpasses bone formation, thereby increasing susceptibility to osteoporosis [3].

Postmenopausal osteoporosis is the most common form of osteoporosis and is strongly associated with estrogen deficiency resulting from ovarian aging [4]. In women, osteoporosis-related fractures represent a major global health burden, with a substantially greater lifetime fracture risk than in men. Estrogen deficiency disrupts bone remodeling by suppressing osteoblast activity while enhancing osteoclastogenesis, thereby accelerating bone loss and increasing skeletal fragility [5]. Mechanistically, estrogen depletion has been associated with dysregulation of the receptor activator of nuclear factor-kappa B ligand (RANKL)/osteoprotegerin (OPG) signaling axis, leading to increased osteoclast differentiation and bone resorption. Elevated RANKL/OPG ratios observed in postmenopausal women with low bone mineral density further support the role of estrogen deficiency in osteoclast activation and skeletal deterioration [6,7].

Osteoporosis constitutes a significant public health problem because of its clinical, social, and economic consequences. Osteoporotic fractures contribute substantially to disability, mortality, reduced quality of life, and healthcare expenditures [8]. Current osteoporosis management includes non-pharmacological and pharmacological approaches. Lifestyle interventions, including adequate nutrition and physical activity, remain important preventive measures. Pharmacological therapies approved for osteoporosis include anabolic agents, antiresorptive agents such as bisphosphonates and denosumab, calcitonin, and selective estrogen receptor modulators (SERMs) [9]. Among SERMs, raloxifene has demonstrated efficacy in postmenopausal osteoporosis by reducing vertebral fracture risk through estrogen receptor modulation [10]. Raloxifene acts as a competitive estrogen agonist in bone tissue, suppressing osteoclastogenesis through regulation of the RANK/RANKL/OPG pathway while preserving osteoblastic activity [11]. However, limitations associated with current therapies, including long-term adverse effects and restricted efficacy [12], have encouraged the exploration of alternative therapeutic strategies and natural products.

In addition to upstream modulation of osteoclastogenesis through the RANK/RANKL/OPG axis, therapeutic approaches targeting downstream mediators of bone resorption have gained increasing attention. Cathepsin K (CatK), a lysosomal cysteine protease highly expressed by activated osteoclasts, is one of the key enzymes responsible for degradation of type I collagen, the principal organic component of bone matrix [13]. Activation of RANK–RANKL signaling stimulates CatK expression, thereby promoting extracellular matrix degradation and bone resorption. Experimental evidence indicates that inhibition of CatK suppresses bone degradation while preserving osteoclast viability and cell–cell communication necessary for skeletal homeostasis. Animal studies involving CatK-deficient models have demonstrated increased bone mass and preserved collagen fibrils, supporting CatK inhibition as a promising antiresorptive strategy for osteoporosis treatment [14].

Natural products have emerged as promising sources of CatK modulators. Several *Artocarpus* species have shown inhibitory effects on CatK expression. Prenylated flavonoids isolated from *Artocarpus altilis* and *Artocarpus heterophyllus* demonstrated CatK inhibitory activity in experimental studies [15], suggesting the therapeutic potential of *Artocarpus*-derived metabolites for osteoporosis management.

Among the less explored species, *Artocarpus integer* (cempedak) contains prenylated flavonoids and chalcone derivatives, including isobavachalcone and morachalcone A, which may contribute to bone-protective effects [16]. Recent phytochemical evidence also suggests the presence of flavonoids with osteogenic and antiresorptive potential. Nevertheless, the anti-osteoporotic activity of *A. integer* leaves remains poorly characterized, particularly regarding their potential to modulate CatK-mediated bone resorption, bone microarchitecture, and osteogenic signaling under estrogen-deficient conditions.

Therefore, this study aimed to investigate the anti-osteoporotic potential of *A. integer* leaf extract in ovariectomized (OVX) rats through an integrated approach involving phytochemical profiling, network pharmacology, biochemical evaluation, bone microarchitectural analysis, and molecular assessment. Liquid chromatography–high-resolution mass spectrometry (LC–HRMS) and network pharmacology were used to identify bioactive constituents and predict osteoporosis-related molecular targets. The in vivo anti-osteoporotic effects were evaluated using femoral calcium content, tibial micro-computed tomography (micro-CT), osteopontin assessment, and RT-PCR analysis of osteoblast- and osteoclast-related genes, including *Runx2*, *Osx*, and *TRAP* using raloxifene as a positive control.

## 2. Materials and Methods

### 2.1. Plant Material Identification

Leaves of *Artocarpus integer* (Thunb.) Merr. (Moraceae) were collected and taxonomically authenticated by Herbarium Depokensis (UIDEP), UI Biota Collection Room, Department of Biology, Faculty of Mathematics and Natural Sciences, Universitas Indonesia. A voucher specimen (No. JI26-P-008) was deposited at the herbarium for future reference.

### 2.2. Sample Preparation

Dried leaves of *A. integer* were ground into a fine powder prior to extraction. Approximately 10 mg of the powdered sample was extracted with 1 mL of MS-grade methanol (Fisher Chemicals, Waltham, MA, USA). The mixture was vortex-mixed at 3000 rpm for 1 min to ensure complete homogenization and then filtered through a 0.20 μm nylon membrane filter. The resulting filtrate was transferred into an HPLC vial and used for LC–HRMS analysis.

### 2.3. Total Flavonoid Content Assay

Total flavonoid content was determined using the aluminum chloride colorimetric method with quercetin as the reference standard. Briefly, quercetin standard solutions were prepared at concentrations of 0, 50, 100, 150, 200, 250, 300, and 350 mg/L. The absorbance of each standard was measured spectrophotometrically, and a calibration curve was constructed. The extract sample was diluted 10-fold, and its absorbance was measured in triplicate at the same wavelength used for the quercetin standards.

### 2.4. LC–HRMS Analysis

Untargeted metabolite profiling was performed using a Vanquish Horizon ultra-high-performance liquid chromatography (UHPLC) system coupled to an Orbitrap Exploris 240 high-resolution mass spectrometer equipped with an OptaMax™ NG heated electrospray ionization (H-ESI) source (Thermo Fisher Scientific, Bremen, Germany).

Chromatographic separation was achieved on an Accucore Phenyl-Hexyl column (100 × 2.1 mm, 2.6 μm; Thermo Fisher Scientific, Vilnius, Lithuania). The mobile phase consisted of water containing 0.1% formic acid (A) and acetonitrile containing 0.1% formic acid (B). Elution was carried out at a flow rate of 0.3 mL min^−1^ over a total run time of 25 min. The gradient program started at 5% B and increased linearly to 90% B over 16 min, followed by a 4 min hold at 90% B before returning to the initial conditions until the end of the run. The column temperature was maintained at 40 °C, and 5 μL of sample was injected for each analysis.

Mass spectrometric data were acquired in both positive and negative ionization modes using Full MS and data-dependent tandem mass spectrometry (dd-MS^2^). Full MS spectra were collected at a resolution of 60,000 FWHM over an *m*/*z* range of 67–1000, with a maximum injection time of 100 ms. MS/MS spectra were acquired at a resolution of 30,000 FWHM using nitrogen as the collision gas and normalized collision energies of 30, 50, and 70.

The H-ESI source was operated with spray voltages of 3500 V and 2500 V in positive and negative ionization modes, respectively. The sheath, auxiliary, and sweep gas flow rates were set at 35, 7, and 1 arbitrary units, respectively. The ion transfer tube and vaporizer temperatures were maintained at 300 °C and 320 °C, respectively.

### 2.5. Data Processing and Metabolite Identification

Raw LC–HRMS data were processed using Compound Discoverer™ version 3.5 (Thermo Fisher Scientific, Waltham, MA, USA). Metabolite annotation was carried out based on accurate mass measurements, isotopic pattern analysis, and comparison of MS/MS fragmentation spectra with entries available in the mzCloud and ChemSpider databases.

To improve compound coverage and annotation confidence, additional screening was performed using specialized flavonoid and natural product databases, including the Arita Lab Flavonoid Database and Natural Products Atlas. Putative metabolite identification was assigned by integrating accurate mass, isotopic distribution, retention behavior, and fragmentation pattern information. A mass accuracy threshold of ≤5 ppm was applied throughout the annotation process.

### 2.6. Network Pharmacology Analysis

Network pharmacology analysis was performed to explore the potential molecular mechanisms underlying the anti-osteoporotic effects of *Artocarpus integer* leaf extract. The phytochemical constituents identified by LC–HRMS were selected as candidate bioactive compounds for target prediction. Compound-related targets were retrieved from public databases, including SwissTargetPrediction, SuperPred, SEA, and/or STITCH, using canonical SMILES or compound names as input. Duplicate targets were removed, and gene symbols were standardized using the UniProt database.

Osteoporosis-associated genes were collected from disease-related databases, including DisGeNET, GeneCards, OMIM, and/or CTD, using the keywords “osteoporosis” and “postmenopausal osteoporosis”. To improve target reliability, disease-associated genes were filtered according to database relevance scores where applicable. All gene symbols were standardized, and duplicate entries were removed.

The predicted compound targets were intersected with osteoporosis-related genes to identify common targets potentially involved in the anti-osteoporotic effects of *A. integer*. The overlapping targets were visualized using a Venn diagram. A compound–target–disease interaction network was constructed using Cytoscape software version 3.10.2. Topological analysis was performed to identify hub genes based on degree, betweenness centrality, and closeness centrality.

Protein–protein interaction (PPI) analysis of the intersecting targets was performed using the STRING database, with the organism set to *Homo sapiens* or *Rattus norvegicus* depending on gene annotation availability. A minimum interaction confidence score of 0.7 was applied to obtain high-confidence interactions. The resulting PPI network was imported into Cytoscape, and clustering analysis was performed using the clusterMaker2 which is Cytoscape 3 version of the clusterMaker plugin with the Markov Cluster Algorithm (MCL). Functional modules were annotated according to their biological relevance to osteoporosis.

Gene Ontology (GO) biological process and Kyoto Encyclopedia of Genes and Genomes (KEGG) pathway enrichment analyses were performed using STRING enrichment, DAVID, Metascape, g, or Enrichr. Enriched terms with false discovery rate (FDR) < 0.05 were considered statistically significant. The major enriched pathways were interpreted in relation to osteoblast differentiation, osteoclastogenesis, estrogen signaling, inflammatory response.

### 2.7. Experimental Animals and Ovariectomy-Induced Osteoporosis Model

Female Sprague–Dawley rats were obtained from IPB University. Animals were healthy, immunocompetent, and not genetically modified. No specific genotype was applicable. Prior to ovariectomy and study initiation, the animals had not been subjected to any experimental procedures or treatments. Animals were housed under standard laboratory conditions (22–25 °C, 12 h light/dark cycle) with free access to a standard pellet diet and drinking water throughout the study.

Experimental osteoporosis was induced by bilateral ovariectomy (OVX). Under general anesthesia, a small dorsal incision was made, and both ovaries were surgically removed. Prior to the surgical procedure, the rats were fasted overnight and anesthetized using a combination of ketamine (70 mg/kg BW) and xylazine (8 mg/kg BW) administered via intraperitoneal injection. Following the bilateral ovariectomy, the muscle layer and skin were sutured. To avoid the confounding systemic effects of antibiotics on the animals’ physiology, no systemic antibiotics were administered. Instead, strict aseptic conditions were maintained throughout the surgery, and topical povidone–iodine was applied daily to the surgical site until complete wound healing and recovery were achieved. Sham-operated animals underwent the same surgical procedure without removal of the ovaries. Following the surgical procedures, all animals were maintained under identical housing conditions for a strict, uniform post-operative recovery and disease-induction period of exactly 28 days (4 weeks) to allow the stable development of estrogen-deficiency-induced bone loss. Experimental treatments for all assigned groups were initiated simultaneously on day 29 post-ovariectomy. To minimize postoperative complications and prevent interference with surgical wound healing, each rat was housed individually following ovariectomy and remained individually housed throughout the experimental period. All animals were maintained under identical environmental conditions, including temperature, humidity, light–dark cycle, diet, and water availability. Treatments and outcome measurements were performed according to a standardized protocol for all groups. No additional measures were implemented to control for cage location effects because each animal was maintained in a separate cage under uniform housing conditions. Animals were randomly allocated to the experimental groups by the investigator responsible for treatment administration; therefore, group allocation was known during the conduct of the experiment. However, laboratory analyses, including biochemical assays, femoral calcium determination, and RT-PCR measurements, were performed using coded samples, and the investigators conducting outcome assessment and statistical analysis were blinded to group allocation until completion of the analyses.

All experimental procedures were approved on 26 February 2026 by the Ethics Committee of the Faculty of Medicine, Universitas Indonesia (No. KET-288/UN2.F1/ETIK/PPM.00.02/2026) and were conducted in accordance with institutional guidelines for the care and use of laboratory animals.

### 2.8. Treatment Protocol

Following acclimatization and postoperative recovery, the animals were randomly assigned to seven experimental groups: (1) sham-operated control (SHAM); (2) ovariectomized control (OVX); (3) OVX rats treated with raloxifene (RAL, 1 mg/kg body weight/day); (4) OVX rats treated with low-dose *A. integer* leaf extract (AIE-L, 12.5 mg/kg body weight/day); (5) OVX rats treated with medium-dose extract (AIE-M, 25 mg/kg body weight/day); and (6) OVX rats treated with high-dose extract (AIE-H, 50 mg/kg body weight/day). Animals were allocated to experimental groups using a computer-generated randomization sequence.

All treatments (the various doses of *Artocarpus integer* leaf extract and the positive control, raloxifene) were administered orally once daily via oral gavage using a gastric intubation tube for a strict, identical duration of 28 days (4 weeks) across all experimental groups. The selected doses of *A. integer* extract were based on previously reported biological activities of flavonoid-rich plant extracts with potential bone-protective properties. The individual rat was considered the experimental unit for all physiological, biochemical, and molecular analyses. Each experimental group consisted of six animals (*n* = 6).

### 2.9. Serum Sample Collection and Adipokine Analysis

At the conclusion of the 8-week treatment period, rats were fasted overnight (12 h) with free access to water. Blood samples were collected via cardiac puncture under anesthesia prior to sacrifice. Whole blood was allowed to clot at room temperature for 30 min in non-anticoagulant tubes and subsequently centrifuged at 3000× *g* for 15 min at 4 °C. The resulting serum was separated, aliquoted, and stored at −80 °C until analysis. Serum concentrations of Leptin, Adiponectin, and Osteopontin were quantified using commercial rat-specific enzyme-linked immunosorbent assay (ELISA) kits (Elabscience, Houston, TX, USA), strictly following the manufacturer’s instructions. Absorbance was measured at 450 nm using a microplate reader (BioTek ELx808, Winooski, VT, USA), and target concentrations were calculated against standard curves.

### 2.10. Determination of Bone Calcium Content by Atomic Absorption Spectrophotometry

Femoral calcium content was designated as the primary outcome measure because it directly reflects bone mineral preservation in the ovariectomized osteoporosis model. Sample size (n=6 per group) was determined based on a power analysis evaluating the ability to detect biologically meaningful differences in femoral calcium content between groups. For the analysis, the whole left femur was carefully harvested from the hind limb of each rat, completely cleared of all adherent muscles and soft tissue, dried, and accurately weighed in its entirety using an analytical balance (with an average dry weight of 240–270 mg before acid digestion. Each whole bone sample was then digested in concentrated nitric acid under controlled heating until complete dissolution was achieved.

Bone calcium content was determined using flame atomic absorption spectrophotometry (FAAS) at the Department of Chemistry, Faculty of Mathematics and Natural Sciences, Universitas Indonesia.

Bone samples were cleaned, dried, and accurately weighed before acid digestion. Samples were digested in concentrated nitric acid under controlled heating until complete dissolution was achieved. The resulting solutions were diluted with deionized water to a known volume and analyzed using an atomic absorption spectrophotometer.

Calcium concentration was measured at 422.7 nm using an air–acetylene flame. Calibration curves were prepared from standard calcium solutions of known concentrations. Quality control samples and reagent blanks were included throughout the analysis. Calcium content was calculated from the calibration curve.

### 2.11. Bone Microarchitecture Analysis with Micro-CT

Isolated tibia bones were fixed in 10% NBF solution for 48 h, then replaced with 70% ethanol and stored at 4 °C until analysis. Scanning was performed with X-ray energy ranging from 40 to 130 kV in single scan mode. The Region of Interest (ROI) was defined in the proximal metaphysis of the tibia, starting 1.0 mm below the growth plate and extending approximately 1.5 mm distally.

Analysis was performed using a Bruker Micro-CT SkyScan 1173 (Kontich, Belgium) High Energy Desktop Micro-CT with a resolution of 10 μm. The obtained three-dimensional images were then analyzed and quantified using image analysis software CTAn (CT-Analyser) (Kontich, Belgium). Parameters measured included bone volume fraction (BV/TV), trabecular thickness (Tb.Th), trabecular number (Tb.N), trabecular separation (Tb.Sp), and total porosity (Po.tot).

### 2.12. Quantitative Real-Time PCR Analysis

Total RNA was isolated from the right femur, representing both trabecular and compact bone tissue. Briefly, after the animals were euthanized, the femur was harvested, thoroughly cleared of all adherent soft tissues and periosteum, and the bone marrow was flushed out using ice-cold phosphate-buffered saline (PBS). The remaining bone tissue was immediately snap-frozen in liquid nitrogen and pulverized into a fine powder using a mortar and pestilence prior to homogenization in Trizol reagent according to the manufacturer’s instructions. RNA concentration and purity were assessed spectrophotometrically, and complementary DNA (cDNA) was synthesized using a reverse transcription kit.

Quantitative real-time PCR (qRT-PCR) was performed to evaluate the expression of genes associated with osteoblast differentiation, osteoclast activity, and estrogen signaling. The target genes included runt-related transcription factor 2 (*Runx2*), osterix (*Osx*), and tartrate-resistant acid phosphatase (*TRAP*). Primer sequences used in this study are presented in Table 1.

Gene expression levels were normalized to a housekeeping gene (β-actin), and relative expression was calculated using the 2^−ΔΔCt^ method. All reactions were performed in triplicate to ensure analytical reliability.

### 2.13. Statistical Analysis

The minimum sample size using Federer’s formula is 4 rats for each of the 6 treatment groups. This is rounded up to six for statistical calculations. Before statistical analysis, data were assessed for normality using the Shapiro–Wilk test and for homogeneity of variance using Levene’s test. Data that satisfied these assumptions were analyzed using one-way ANOVA followed by Tukey’s multiple comparison test. If the assumptions of normality or homogeneity of variance were not met, appropriate non-parametric analyses (Kruskal–Wallis test followed by Dunn’s post hoc test) were applied. Statistical significance was considered at *p* < 0.05.

## 3. Results

### 3.1. The Total Flavonoid Content of the Extract

The total flavonoid content of the extract, determined by the aluminum chloride colorimetric method using quercetin as the reference standard, was 161.8 ± 16.7 mg QE/g extract (equivalent to 16.18 ± 1.67% *w*/*w* QE). The quercetin calibration curve showed good linearity over the concentration range of 0–350 mg/L $\left(y=0.0063x+0.0723,R^{2}=0.9939\right)$.

### 3.2. LC–HRMS Profiling of Phytochemical Constituents in Artocarpus integer Leaf Extract

**Figure 1:**
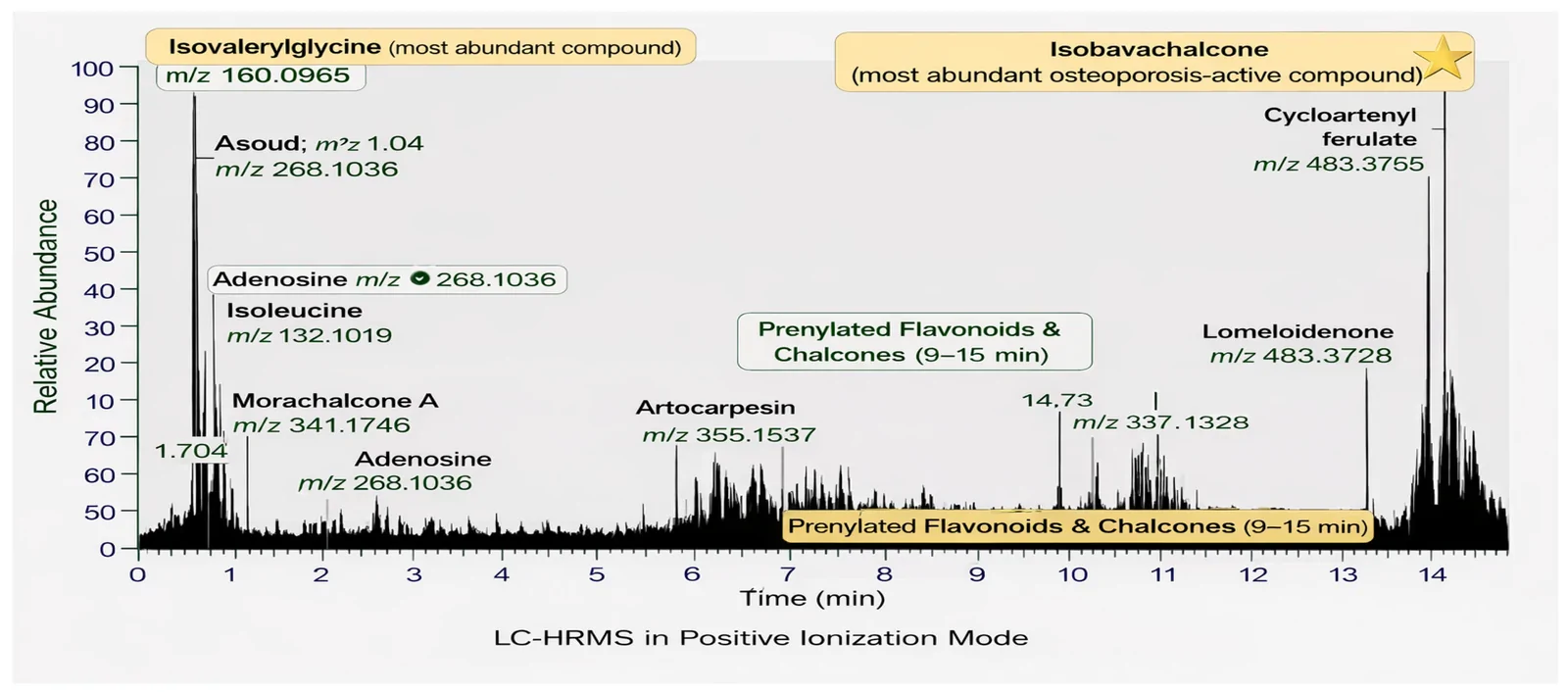
Total Ion Chromatogram (TIC MS) profiling the chemical constituents of the *Artocarpus integer* (Cempedak) leaf extract over a 25 min run time. The 14 numbers pinpoint the specific chromatographic peaks corresponding to the 14 major identified compounds characterized in Table 2 (Compounds **1**–**14**), ordered by their respective retention times (RT).

Untargeted liquid chromatography–high-resolution mass spectrometry (LC–HRMS) analysis was performed to characterize the phytochemical composition of *Artocarpus integer* leaf extract. The total ion chromatogram (TIC) acquired in positive electrospray ionization mode (ESI^+^) demonstrated the presence of metabolites with distinct chromatographic behaviors over a retention time range of 0–25 min (Figure 1). Early eluting peaks (retention time (RT) < 2 min) corresponded predominantly to highly polar metabolites, whereas peaks detected between 5 and 15 min represented moderately polar secondary metabolites, including flavonoids and chalcone derivatives. Peaks eluting after 15 min were associated with less polar constituents.

Several metabolites were tentatively identified based on accurate mass measurement, isotopic pattern, retention time, and database matching using mzCloud (HighChem, Bratislava, Slovakia) and ChemSpider (Cambridge, UK). Major phytochemicals included morachalcone A (RT 6.40 min, *m/z* 341.1747), artocarpesin (RT 9.13 min, *m/z* 355.1540), and isobavachalcone (RT 10.48 min, *m/z* 325.1434), which belong to prenylated chalcone and flavonoid classes (Table 2). In addition, primary metabolites such as adenosine and isovalerylglycine were detected at earlier retention times.

Among the detected metabolites, isovalerylglycine exhibited the highest peak intensity overall, indicating its relative abundance within the extract. Among compounds associated with bone-related pharmacological activity, isobavachalcone showed the highest relative abundance based on chromatographic peak area. The identification of prenylated flavonoids and chalcone derivatives in *A. integer* leaves suggests the presence of bioactive constituents previously reported to regulate bone metabolism, including osteoblast differentiation and osteoclast activity.

Among the compounds, isobavachalcone (RT 10.48 min, *m*/*z* 325.1434) was identified as the most abundant anti-osteoporosis compound, while isovalerylglycine (RT 0.72 min, *m*/*z* 160.0965) was the most abundant overall metabolite. Prenylated flavonoids such as artocarpesin and chalcone derivatives such as morachalcone A are known to contribute to osteogenic activity through the stimulation of osteoblast differentiation and inhibition of osteoclastogenesis.

### 3.3. Network Pharmacology Analysis of Artocarpus integer Compounds Against Osteoporosis

Network pharmacology analysis demonstrated that the 14 identified compounds of *Artocarpus integer* interacted with multiple osteoporosis-related genes involved in bone remodeling, inflammatory signaling, hormonal regulation, and metabolic pathways. Isobavachalcone and morachalcone A exhibited the greatest target overlap with osteoporosis-related genes (37 targets each), followed by artocarpesin (33 targets), isovalerylglycine (22 targets), and adenosine (15 targets). Two genes, *Dpp4* and *Ca2*, were shared among all five compounds, indicating their potential roles as central targets in the anti-osteoporosis network.

Several genes associated with osteoclast activity and extracellular matrix remodeling, including *Mmp2*, *Src*, and *Ptgs2*, were shared among multiple compounds, suggesting possible regulation of bone resorption and inflammatory bone remodeling. Genes involved in estrogen signaling and postmenopausal osteoporosis, including *Esr1*, *Esr2*, and *Cyp19a1*, were also identified, indicating potential modulation of estrogen-deficiency-related bone loss. Furthermore, metabolic and osteogenic regulators such as *Igf1r*, *Gsk3b*, *Pparg*, and *Sirt1* were identified, suggesting possible effects on osteoblast differentiation and skeletal homeostasis.

**Figure 2:**
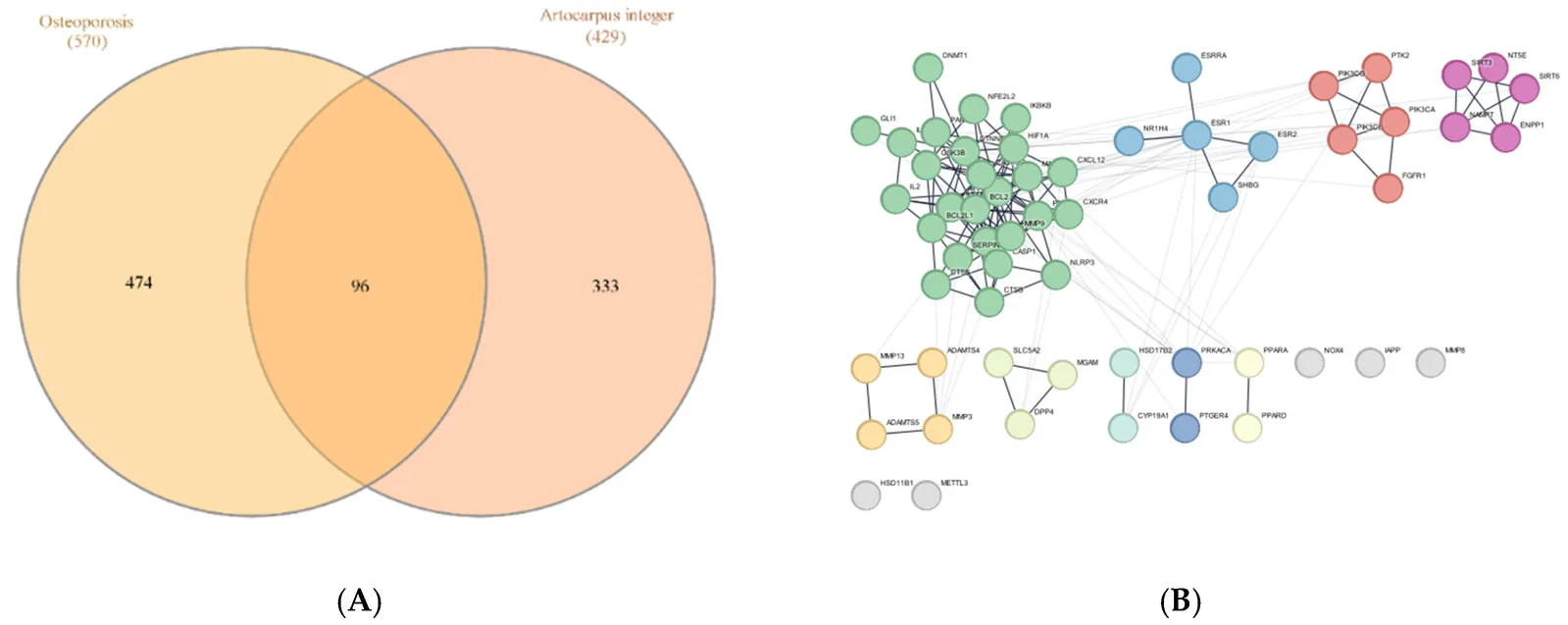
Identification and clustering analysis of common targets between *Artocarpus integer* phytochemicals and osteoporosis-related genes. (**A**) Venn diagram showing the overlap between osteoporosis-associated targets and predicted targets of *Artocarpus integer* phytochemicals, resulting in 96 common targets. (**B**) Functional clustering analysis of the intersecting targets generated using Cytoscape and CytoCluster is Cytoscape 3.0.X., illustrating major gene modules associated with estrogen signaling, osteogenesis, osteoclast differentiation, inflammation, extracellular matrix remodeling, oxidative stress, and metabolic regulation.

As shown in Figure 2, the Venn diagram shows the overlap between osteoporosis-related target genes and predicted targets of *Artocarpus integer* phytochemicals. A total of 570 osteoporosis-associated targets and 429 predicted targets of *A. integer* phytochemicals were collected, yielding 96 intersecting genes potentially involved in the anti-osteoporotic activity of *A. integer*.

MCL clustering analysis was performed in Cytoscape using the clusterMaker2 is the Cytoscape 3 version of the clusterMaker plugin to identify functional modules among intersecting osteoporosis-related targets. Functional annotation of clusters was curated based on known biological roles in osteogenesis, osteoclastogenesis, inflammatory signaling, extracellular matrix remodeling, estrogen signaling, metabolic regulation, and oxidative stress associated with osteoporosis.

The network pharmacology analysis revealed that the intersecting osteoporosis-related targets of *Artocarpus integer* phytochemicals were organized into several functional modules that collectively converge into four major anti-osteoporotic mechanisms.

As shown in Table 3, the first mechanism involves the suppression of osteoclastogenesis and inflammatory signaling, represented by Clusters 1 and 5, which include genes associated with inflammasome activation, inflammatory cytokine signaling, and extracellular matrix degradation, such *As Nlrp3*, *Casp1*, *Il6*, *Ptgs2*, *Ctsb*, *Ctss*, *Mmp3*, *Mmp9*, *Mmp13*, and *Ikbkb*. These targets suggest a potential inhibitory effect on osteoclast activation and bone resorption.

The second mechanism is related to estrogen signaling and steroid hormone regulation, represented by Clusters 2 and 9, including *Esr1*, *Esr2*, *Esrra*, *Shbg*, *Cyp19a1*, and *Hsd17b2.* These genes are strongly associated with postmenopausal osteoporosis and indicate a potential role of *A. integer* phytochemicals in modulating estrogen-deficiency-related bone loss.

The third mechanism involves osteoblast differentiation, bone metabolism, and mineralization, represented by Clusters 3 and 4, which include genes involved in anabolic and osteogenic signaling pathways, such as *Fgfr1*, *Pik3ca*, *Pik3cd*, *Pik3cg*, *Ptk2*, *Sirt3*, *Sirt6*, *Nampt*, *Nt5e*, and *Enpp1.* These targets are implicated in osteoblast survival, skeletal mineral homeostasis, and bone formation.

The fourth mechanism is associated with the metabolic–bone axis and lipid metabolism, represented by Clusters 6–8, including *Dpp4*, *Slc5a2*, *Mgam*, *Ppara*, *Ppard*, *Prkaca*, and *Ptger4*, suggesting that *A. integer* may regulate bone remodeling through metabolic and hormonal pathways linked to obesity and glucose homeostasis.

Importantly, these predicted mechanisms closely correspond to the selected RT-PCR biomarkers used for molecular validation. *Runx2* and *Osx (SP7*), which regulate osteoblast differentiation and mineralization, are biologically linked to osteogenic signaling modules (Clusters 3–4) involving *Fgfr1*, *Pik3ca*, *Ptk2*, *Sirt3*, and *Enpp1*. In contrast, *Trap*, *Ctsk*, and *Nfatc1*, key markers of osteoclast differentiation and bone resorption, are associated with inflammatory and osteoclastogenic modules (Clusters 1 and 5) enriched with *Il6*, *Ptgs2*, *Ctsb*, *Ctss*, *Mmp3*, *Mmp9*, *Mmp13*, *Nlrp3*, and *Casp1*. Furthermore, *Esr1*, included in the RT-PCR panel, directly corresponds to the estrogen signaling modules (Clusters 2 and 9) and provides mechanistic validation of estrogen-related regulation in ovariectomy-induced osteoporosis. After enrichment, the primary pathway has 5 pathways as shown in Table 4.

Overall, the integration of network pharmacology and RT-PCR analysis provides a coherent mechanistic framework in which *A. integer* phytochemicals may exert anti-osteoporotic effects through simultaneous enhancement of osteoblast activity, suppression of osteoclastogenesis, restoration of estrogen-related signaling, and the regulation of metabolic pathways associated with bone remodeling.

### 3.4. Effects of Artocarpus integer Leaf Extract on Metabolic, Hormonal, and Bone-Related Parameters in Ovariectomized Rats

**Figure 3:**
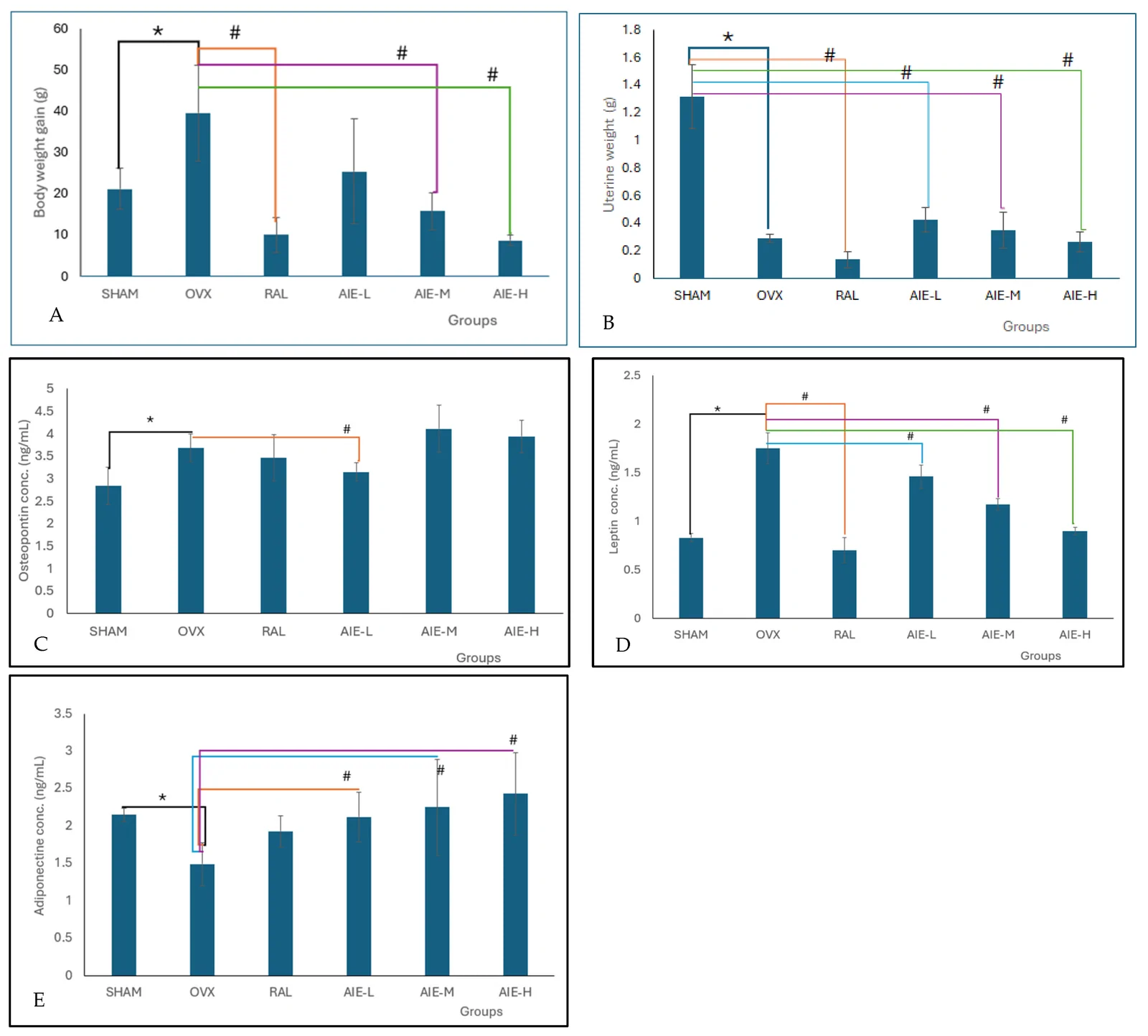
Effects of *Artocarpus integer* Leaf Extract on Physiological and Biochemical Parameters in Ovariectomized Rats. (**A**). Body weight; (**B**). Uterine weight; (**C**). Osteopontin concentration; (**D**). Leptin; (**E**). Adiponectine. (Serum samples were collected at terminal sacrifice following an overnight fast, and biomarker concentrations [Leptin, Adiponectin, and Osteopontin] were measured using ElabscienceELISA kits (Wuhan, Hubei, China). Note: Data are expressed as mean ± SD (*n* = 6). * p<0.05 vs. SHAM group; # p<0.05 vs. Negative Control group using one-way ANOVA followed by Tukey’s post hoc test.

The effects of *Artocarpus integer* leaf extract on body weight gain, uterine weight, serum osteopontin, leptin, adiponectin, and femoral calcium content in ovariectomized rats are presented in Figure 3.

OVX rats exhibited a marked increase in body weight gain compared to the sham-operated group (39.5 ± 11.6 vs. 21.1 ± 4.9 g). Treatment with raloxifene substantially reduced body weight gain to 9.9 ± 4.1 g. Similarly, administration of *A. integer* leaf extract attenuated OVX-induced weight gain in a dose-dependent manner, with the greatest reduction observed in the high-dose group (AIE-H; 8.7 ± 1.2 g), followed by the medium-dose group (AIE-M; 15.7 ± 4.4 g).

A significant decrease in uterine weight was observed in OVX rats compared to sham-operated animals (0.29 ± 0.03 vs. 1.32 ± 0.23 g), confirming successful induction of estrogen deficiency. Treatment with *A. integer* extract resulted in uterine weights ranging from 0.27 ± 0.07 to 0.43 ± 0.09 g, which remained lower than those of the sham group.

Serum osteopontin concentrations were elevated following ovariectomy, increasing from 2.84 ± 0.52 ng/mL in the sham group to 3.68 ± 0.57 ng/mL in the OVX group. Among the treatment groups, the lowest osteopontin concentration was observed in the AIE-L group (3.15 ± 0.81 ng/mL), whereas higher levels were recorded in the AIE-M (4.11 ± 1.02 ng/mL) and AIE-H (3.94 ± 1.36 ng/mL) groups.

Serum leptin levels increased markedly in OVX rats (1.75 ± 0.16 ng/mL) compared with sham controls (0.83 ± 0.04 ng/mL). Raloxifene treatment reduced leptin concentrations to 0.70 ± 0.13 ng/mL. Likewise, *A. integer* extracts decreased leptin levels in a dose-dependent manner, from 1.46 ± 0.12 ng/mL in the AIE-L group to 0.90 ± 0.04 ng/mL in the AIE-H group.

In contrast, adiponectin concentrations were significantly reduced following ovariectomy (1.49 ± 0.29 ng/mL) relative to the sham group (2.15 ± 0.09 ng/mL). Treatment with *A. integer* extract increased adiponectin levels in a dose-dependent manner, reaching 2.43 ± 0.55 ng/mL in the AIE-H group. A similar improvement was observed in the raloxifene-treated group (1.93 ± 0.21 ng/mL).

Table 5 showed that Femoral calcium content was significantly lower in OVX rats than in sham-operated animals (43.02 ± 5.42 vs. 59.77 ± 8.02; *p* < 0.05), indicating bone mineral loss associated with estrogen deficiency. Treatment with *A. integer* extract increased femoral calcium content in all treatment groups compared to untreated OVX rats. The highest calcium content was observed in treated group is the AIE-M group (63.11 ± 12.88), exceeding the values recorded in raloxifene-treated groups.

Overall, ovariectomy induced adverse changes in metabolic and bone-related parameters, including increased body weight gain, elevated leptin and osteopontin levels, reduced adiponectin concentrations, uterine atrophy, and decreased femoral calcium content. Treatment with *A. integer* leaf extract ameliorated several of these alterations, particularly by reducing body weight gain and leptin levels, increasing adiponectin concentrations, and preserving femoral calcium content, with the most pronounced effects generally observed in the medium-dose groups.

### 3.5. Quantitative and Qualitative Analysis of Micro-CT Parameters

**Figure 4:**
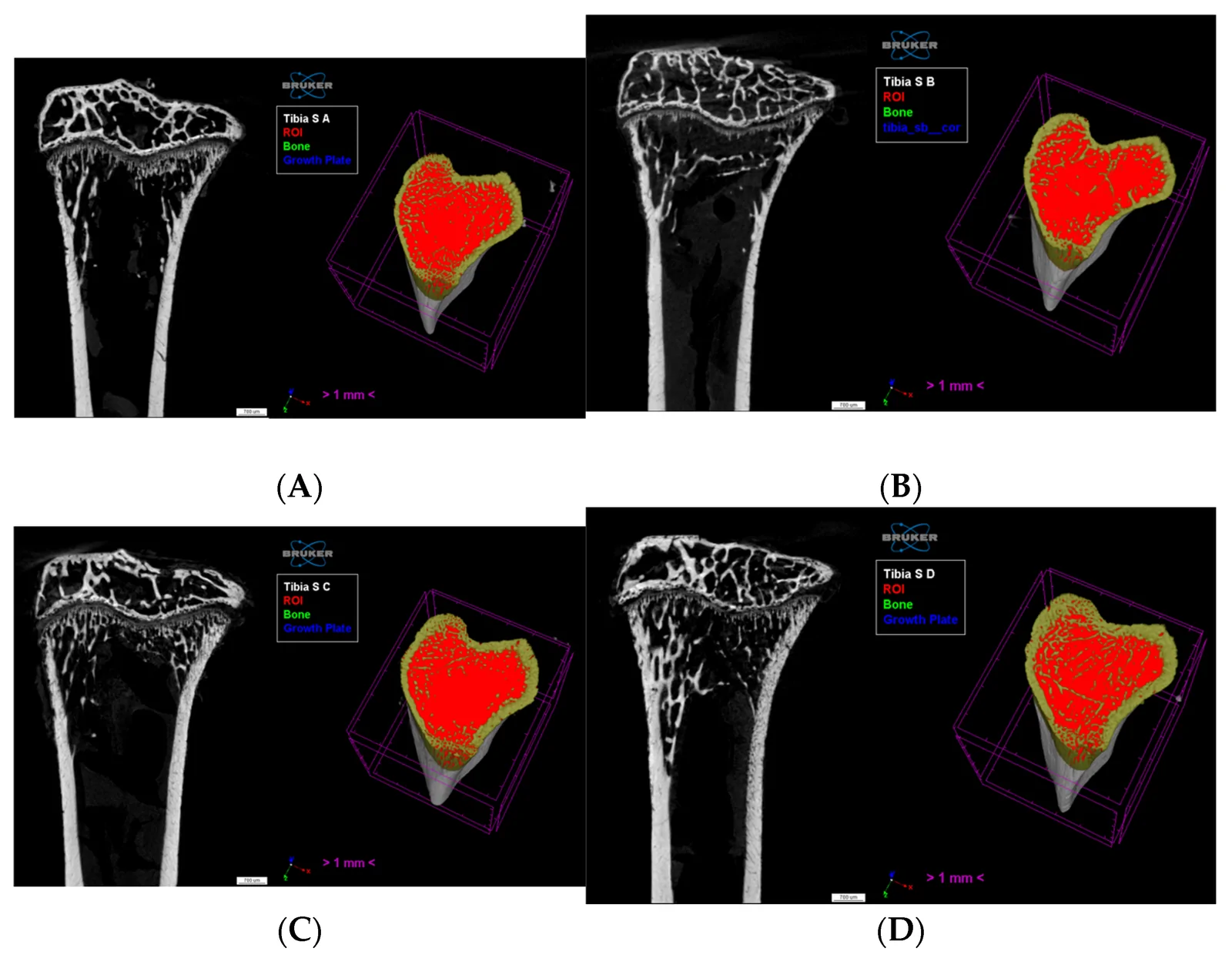
Qualitative micro-CT 3D reconstructions of proximal tibia architecture following 28 days of daily oral treatment. Coronal sections and three-dimensional reconstructions of trabecular bone from (**A**) Sham, (**B**) ovariectomized (OVX), (**C**) raloxifene-treated, and (**D**) high-dose *Artocarpus integer* extract (AIE-H) groups. Ovariectomy induced the deterioration of trabecular bone characterized by reduced trabecular connectivity and enlarged marrow spaces. Treatment with raloxifene and AIE-H preserved trabecular architecture, with AIE-H showing a dense trabecular network comparable to the Sham group. Red (ROI—Region of Interest): Highlights the specific internal volume selected for 3D analysis (the trabecular bone compartment being measured inside the tibia).Green (Bone): Indicates the segmented threshold for total bone tissue structure (such as the outer cortical shell/cortex surrounding the interior region).Purple Box Lines: Represents the 3D bounding box frame of the reconstructed dataset, with the purple axis arrows (x,y,z) in the lower-left corner indicating spatial orientation and scale (>1 mm<).

To evaluate the effects of *Artocarpus integer* leaf extract on bone microarchitecture under estrogen-deficient conditions, qualitative 3D micro-computed tomography (micro-CT) profiling was paired with quantitative trabecular histomorphometric analysis (Figure 4 and Table 6).

Qualitatively, the Sham group displayed a highly dense, well-connected, and uniform trabecular network within the proximal tibial metaphysis (Figure 4A). Conversely, bilateral ovariectomy led to severe architectural failure in the Negative Control group, characterized by massive resorption voids, extensive thinning, and a clear loss of structural trabecular connectivity (Figure 4B). This structural degradation was distinctly mitigated in both the Raloxifene (Figure 4C) and high-dose *Artocarpus integer* extract (AIE-H, Figure 4D) treatment groups, both of which preserved a dense and interconnected structural matrix.

Representative micro-computed tomography (micro-CT) images of the proximal tibia are shown in Figure 4. The Sham group exhibited a dense and well-connected trabecular network with thick trabeculae and limited marrow spaces. In contrast, ovariectomy resulted in marked deterioration of trabecular architecture, characterized by reduced trabecular connectivity, enlarged marrow cavities, and thinning of the trabecular network. Raloxifene treatment partially restored trabecular organization and connectivity. Similarly, high-dose *Artocarpus integer* extract (AIE-H) markedly preserved trabecular microarchitecture, exhibiting a dense trabecular network and improved structural integrity compared with the OVX group. These qualitative observations are consistent with the morphometric analysis, in which AIE-H increased bone volume fraction (BV/TV) and trabecular thickness (Tb.Th) while reducing total porosity.

These quantitative metrics strictly corroborate the qualitative findings. Ovariectomy triggered a severe drop in structural parameters, reducing trabecular thickness (Tb.Th) to a range of 0.180−0.199 mm and reducing the overall trabecular number (Tb.N) down to $1.58-1.67{\;\mathrm{mm}}^{-1}$ in the untreated Negative Control group.

Intervention with AIE-H successfully counteracted this bone loss, maintaining a bone volume fraction (BV/TV) of up to 43.43% and actively increasing trabecular thickness to values between 0.231 mm and 0.259 mm, which matched or outpaced the performance of the positive control, Raloxifene. Taken together, these metrics confirm that *A. integer* extract exerts a profound protective effect against estrogen-deficiency-induced osteoporotic microarchitectural deterioration.

### 3.6. Effects of Artocarpus integer Leaf Extract on Osteogenic and Osteoclastogenic Gene Expression

To validate the molecular targets predicted by network pharmacology analysis, the expression of genes associated with osteoblast differentiation (*Runx2* and *Osx*), and osteoclastogenesis (*Trap*), were evaluated using quantitative RT-PCR as shown in Table 7.

As expected, ovariectomy resulted in downregulation of the osteogenic markers *Runx2* and *Osx* compared to the sham group, indicating impaired osteoblast differentiation under estrogen-deficient conditions. In contrast, expression of the osteoclast-related genes *TRAP* was increased in OVX rats, reflecting enhanced osteoclast activity and bone resorption.

Treatment with raloxifene and *A. integer* leaf extract reversed these changes. The treated groups showed increased expression of *Runx2* and *Osx* and reduced expression of *Trap* relative to the OVX group.

These findings are consistent with the network pharmacology predictions and indicate that *A. integer* may exert anti-osteoporotic effects through stimulation of osteoblast differentiation, suppression of osteoclastogenesis, and modulation of estrogen signaling.

## 4. Discussion

### 4.1. Phytochemical Profile of Artocarpus integer Leaf Extract Determined by LC–HRMS

LC–HRMS analysis revealed that *A. integer* leaf extract contains diverse bioactive compounds, including prenylated flavonoids, chalcones, catechins, and phytoestrogenic flavonoids such as genistein, apigenin, luteolin, and naringenin. These compounds have previously been reported to regulate osteoblast differentiation, inhibit osteoclastogenesis, and reduce oxidative stress, suggesting potential relevance to bone health [17,18,19,20].

Taken together, the LC–HRMS results suggest that the biological activity of *A. integer* is likely attributable to the combined action of multiple phytochemical constituents rather than a single active compound. The coexistence of prenylated flavonoids, chalcones, catechins, and other polyphenolic metabolites may provide synergistic effects on pathways involved in osteogenesis, osteoclastogenesis, inflammation, oxidative stress, and estrogen signaling. This multi-component nature is consistent with the complex pathophysiology of osteoporosis and provides a strong rationale for the subsequent network pharmacology analysis conducted in this study. The identified compounds were predicted to interact with numerous osteoporosis-related targets, supporting the hypothesis that *A. integer* exerts its anti-osteoporotic effects through a multi-target mechanism involving both bone-forming and bone-resorbing pathways.

### 4.2. Prospective In Silico Mechanisms and Future Directions

To explore the prospective molecular mechanisms by which the tentatively identified constituents of *Artocarpus integer* might exert their osteoprotective effects, an in silico network pharmacology analysis was conducted (Figure 2, Table 3 and Table 4). It is important to emphasize that because these bioactive compounds were profiled via mass spectrometry without physical fraction isolation, and because direct pathway validation (such as Western blotting or knockout models) was outside the scope of the current study, these network interactions remain predictive.

However, this integrated analysis provides a valuable theoretical framework. Ultimately, these in silico pathway nodes serve as a prospective roadmap, highlighting critical targets for future functional studies to definitively validate the exact molecular crosstalk involved.

Osteoporosis is a complex disease involving multiple biological processes, including impaired osteoblast activity, excessive osteoclast-mediated bone resorption, chronic inflammation, oxidative stress, and hormonal imbalance [21]. Because of this complexity, single-target therapies may not fully address the underlying mechanisms driving bone loss. In the present study, network pharmacology was employed to explore the potential molecular mechanisms through which *Artocarpus integer* phytochemicals may exert anti-osteoporotic effects. By integrating compounds identified through LC–HRMS with osteoporosis-associated targets, a network of intersecting genes was established, providing insight into the biological pathways potentially affected by the extract.

The compound–target network revealed several highly connected hub genes, including *Esr1*, *Esr2*, *Cyp19a1*, *Akt1*, *Igf1r*, *Gsk3b*, *Ptgs2*, *Mmp2*, *Mmp9*, *Dpp4*, and *Nlrp3.* These genes are known to play important roles in bone remodeling and collectively suggest that *A. integer* may influence both bone formation and bone resorption [22,23]. Rather than acting through a single pathway, the identified phytochemicals appear to interact with multiple molecular targets simultaneously, which is consistent with the multi-component nature of plant extracts and the multifactorial pathogenesis of osteoporosis.

Cluster analysis further demonstrated that many of the intersecting genes were associated with inflammatory and osteoclastogenic processes. Notably, genes such as *Nlrp3*, *Casp1*, *Il6*, *Ptgs2*, *Mapk1*, *Mapk14*, *Ctsb*, *Ctss*, and *Mmp9* formed a major functional module linked to inflammation-mediated bone resorption [24,25]. Increasing evidence indicates that chronic low-grade inflammation contributes substantially to postmenopausal osteoporosis. Estrogen deficiency promotes the production of pro-inflammatory cytokines, including IL-6 and TNF-α, which stimulate osteoclast differentiation and activity through activation of NF-κB and MAPK signaling pathways [26]. The presence of these inflammatory targets within the network suggests that *A. integer* may help attenuate bone loss by suppressing inflammatory signaling and limiting excessive osteoclast activation.

Another important finding was the enrichment of genes involved in estrogen signaling and steroid hormone metabolism, including *Esr1*, *Esr2*, *Esrra*, *Cyp19a1*, *Shbg*, and *Hsd17b2*. Estrogen plays a central role in maintaining skeletal homeostasis, and its decline following menopause is one of the primary causes of osteoporosis [27]. The identification of estrogen-related targets is particularly interesting because several compounds detected in *A. integer*, such as genistein, luteolin, and apigenin, have previously been reported to exhibit phytoestrogenic activity [28]. These compounds may partially compensate for estrogen deficiency by interacting with estrogen receptors and modulating downstream signaling pathways involved in bone turnover. Such effects could contribute to the preservation of bone mass and suppression of excessive osteoclastogenesis under estrogen-deficient conditions.

In addition to pathways associated with bone resorption, the network highlighted several genes involved in osteoblast differentiation and bone formation. Targets such as *Fgfr1*, *Igf1r*, *Akt1*, *Pik3ca*, *Ptk2*, *Gsk3b*, and *Ctnnb1* are closely associated with osteogenic signaling pathways [29], particularly PI3K–Akt and Wnt/β-catenin signaling. These pathways are essential for osteoblast proliferation, differentiation, and survival. Activation of PI3K–Akt signaling promotes osteogenesis partly through the regulation of key transcription factors such as *Runx2* and *Osx* [30], whereas Wnt/β-catenin signaling is critical for maintaining bone formation and trabecular integrity [31]. The enrichment of these pathways suggests that *A. integer* may support bone formation in addition to suppressing bone resorption, thereby restoring the balance of bone remodeling disrupted by estrogen deficiency.

The network analysis also identified genes involved in extracellular matrix remodeling, including *Mmp1*, *Mmp2*, *Mmp3*, *Mmp9*, *Mmp13*, *Ctsb*, and *Ctss*. Matrix metalloproteinases and cathepsins are important mediators of bone matrix degradation during osteoclastic resorption [32]. Elevated expression of these enzymes has been associated with accelerated bone turnover and deterioration of trabecular microarchitecture in osteoporosis. Therefore, modulation of matrix remodeling pathways may represent another mechanism through which *A. integer* contributes to skeletal preservation.

Oxidative stress emerged as an additional biological process potentially affected by the extract. Targets such as *Nfe2l2 (Nrf2)*, *Sirt3*, *Sirt6*, *Nampt*, and *Enpp1* suggest involvement of antioxidant defense pathways [33]. Oxidative stress is increasingly recognized as a contributor to osteoporosis because excessive reactive oxygen species impair osteoblast function while promoting osteoclast differentiation [21]. Activation of *Nrf2*- and sirtuin-related pathways has been shown to protect bone cells from oxidative damage and maintain skeletal homeostasis [34]. The presence of these targets is consistent with the identification of antioxidant-rich phytochemicals, including catechins, mangiferin, luteolin, and EGCG, in the LC–HRMS analysis.

Interestingly, several targets were also linked to metabolic regulation, including *Dpp4*, *Ppara*, *Ppard*, and *Enpp1* [35]. These genes connect energy metabolism, obesity, and skeletal health, highlighting the growing recognition of the bone–metabolism axis. Because ovariectomy often induces weight gain and metabolic alterations, regulation of these pathways may contribute indirectly to improved bone health. This observation is particularly relevant given the changes in body weight observed in the OVX animals and supports the hypothesis that *A. integer* may exert beneficial effects beyond direct modulation of bone cells.

The biological significance of the network was further supported by GO and KEGG enrichment analyses, which identified pathways related to osteoclast differentiation, estrogen signaling, PI3K–Akt signaling, MAPK signaling, Wnt signaling, NF-κB signaling, extracellular matrix organization, and oxidative stress responses. Together, these pathways provide a coherent mechanistic explanation for how the phytochemicals present in *A. integer* may influence bone remodeling under estrogen-deficient conditions.

Importantly, the network pharmacology findings showed strong agreement with the experimental endpoints selected for biological validation. Osteogenic pathways identified in the network are closely associated with the RT-PCR markers *Runx2* and *Osx*, whereas inflammatory and osteoclastogenic pathways correspond to *Trap*. Taken together, these findings suggest that the anti-osteoporotic activity of *A. integer* is likely mediated through coordinated regulation of osteoblast differentiation, osteoclast suppression, estrogen signaling, inflammation, oxidative stress, and extracellular matrix remodeling. This multi-target mode of action is consistent with the complex nature of both osteoporosis and plant-derived phytochemical mixtures.

### 4.3. Effects of Artocarpus integer on Physiological, Biochemical, and Bone-Related Parameters in Ovariectomized Rats

The ovariectomized (OVX) rat model is widely used to mimic postmenopausal osteoporosis because it reproduces many of the physiological, metabolic, and skeletal alterations associated with estrogen deficiency [6,36,37,38]. In the present study, OVX rats exhibited increased body weight gain, uterine atrophy, elevated serum leptin and osteopontin concentrations, reduced adiponectin levels, and decreased femoral calcium content compared with sham-operated animals. These findings are consistent with the established consequences of estrogen deprivation and confirm the successful induction of osteoporosis.

One of the most prominent effects of ovariectomy was the increase in body weight gain. Estrogen plays a central role in regulating energy homeostasis, lipid metabolism, and adipose tissue distribution [39]. Consequently, estrogen deficiency often leads to increased adiposity and metabolic dysfunction [40]. The substantial weight gain observed in OVX rats is consistent with previous studies demonstrating that loss of ovarian function promotes fat accumulation and metabolic disturbances [41]. Increased adiposity may also negatively affect bone health through the production of pro-inflammatory cytokines and adipokines that influence bone remodeling [42].

Treatment with *A. integer* extract attenuated OVX-induced weight gain, particularly in the medium- and high-dose groups. The high-dose extract produced weight gain comparable to that observed in raloxifene-treated animals, suggesting that the extract may partially counteract metabolic alterations associated with estrogen deficiency. These effects may be attributed to bioactive constituents identified by LC–HRMS, including genistein, luteolin, naringenin, artocarpesin, and isobavachalcone, which have been reported to possess anti-inflammatory, phytoestrogenic, and metabolic regulatory activities.

A marked reduction in uterine weight was observed following ovariectomy, reflecting the loss of estrogenic stimulation of reproductive tissues. Uterine atrophy is a well-established indicator of successful ovariectomy and confirms the estrogen-deficient status of the animals [6,43]. Although *A. integer* treatment slightly increased uterine weight compared with untreated OVX rats, the values remained substantially lower than those observed in sham-operated animals. These findings suggest that the extract does not exert strong uterotrophic activity. Such a profile is desirable because an ideal anti-osteoporotic intervention should preserve skeletal health without excessive stimulation of estrogen-sensitive reproductive tissues.

The metabolic effects of ovariectomy were further reflected by alterations in adipokine concentrations [44]. Serum leptin levels were markedly elevated in OVX rats, whereas adiponectin concentrations were significantly reduced. Leptin is primarily secreted by adipose tissue and is often elevated under conditions of increased adiposity and estrogen deficiency [45]. Excessive leptin production has been associated with chronic inflammation and dysregulated bone remodeling. In contrast, adiponectin exhibits anti-inflammatory and insulin-sensitizing properties and has been linked to improved metabolic homeostasis and skeletal health [46]. Treatment with *A. integer* decreased leptin levels while increasing adiponectin concentrations in a dose-dependent manner, indicating favorable modulation of the adipose–bone axis. The simultaneous reduction in leptin and elevation in adiponectin suggest that the extract may alleviate metabolic disturbances associated with estrogen deficiency while creating a more favorable systemic environment for bone maintenance.

Serum osteopontin concentrations were also elevated following ovariectomy, supporting the presence of increased skeletal turnover under estrogen-deficient conditions [47]. Osteopontin is a multifunctional extracellular matrix protein involved in osteoblast–osteoclast communication, cell adhesion, inflammation, and bone remodeling. Elevated osteopontin levels have frequently been associated with enhanced bone turnover and inflammatory bone loss [48]. Although treatment with *A. integer* produced variable effects on osteopontin concentrations and did not show a clear dose-dependent trend, this finding should be interpreted cautiously. Osteopontin participates in both bone formation and bone resorption, and its expression may increase during active skeletal remodeling and matrix repair. Therefore, osteopontin may reflect dynamic remodeling activity rather than serving as a direct indicator of bone deterioration or protection [49].

In addition to metabolic and biochemical alterations, ovariectomy resulted in a marked reduction in femoral calcium content, indicating loss of bone mineral following estrogen deficiency [50]. Estrogen plays a critical role in maintaining calcium homeostasis by regulating osteoblast and osteoclast activity as well as calcium absorption and retention. Under estrogen-deficient conditions, enhanced osteoclastic bone resorption accelerates mineral loss from the skeletal matrix [51]. The decreased femoral calcium content observed in OVX rats therefore supports the occurrence of osteoporosis-like changes and is consistent with the observed physiological and biochemical abnormalities.

Treatment with *A. integer* increased femoral calcium content in all treatment groups compared with untreated OVX rats, with the most pronounced effect observed in the medium-dose group. Interestingly, femoral calcium content in this group exceeded that of both the sham-operated and raloxifene-treated animals, suggesting substantial preservation of skeletal mineralization. Although calcium content alone cannot fully describe bone quality or microarchitecture, it remains an important indicator of bone mineral status and skeletal integrity.

The improvement in calcium content may be associated with the diverse phytochemicals identified in the extract, particularly prenylated flavonoids and chalcone derivatives such as artocarpesin, isobavachalcone, and morachalcone A, together with phytoestrogenic flavonoids including genistein, apigenin, luteolin, and naringenin [52]. These compounds have been reported to stimulate osteoblast differentiation, suppress osteoclast activity, and enhance bone formation through modulation of estrogen signaling, PI3K–Akt signaling, and Wnt/β-catenin pathways.

The network pharmacology analysis further supports these observations. Several key targets associated with metabolism, estrogen signaling, and bone remodeling, including ESR1, ESR2, IGF1R, AKT1, GSK3B, CTNNB1, DPP4, PTGS2, and NLRP3, were identified as potential targets of *A. integer* phytochemicals. Modulation of these pathways may contribute to the observed improvements in body weight gain, adipokine balance, and bone mineral preservation. Furthermore, the predicted interactions between phytochemicals and estrogen-related signaling pathways suggest that the extract may partially compensate for estrogen deficiency while simultaneously regulating inflammatory and metabolic processes.

Taken together, the physiological, biochemical, and bone-related findings demonstrate that *A. integer* ameliorated several adverse consequences of estrogen deficiency. The extract reduced body weight gain, improved adipokine profiles, preserved femoral calcium content, and partially normalized biomarkers associated with metabolic and skeletal homeostasis. These effects are consistent with the multi-target mechanisms predicted by network pharmacology and suggest that *A. integer* may exert anti-osteoporotic activity through coordinated regulation of metabolism, inflammation, estrogen signaling, and bone remodeling. Further validation through microCT analysis and RT-PCR assessment of osteogenic and osteoclastogenic markers will provide deeper insight into the molecular mechanisms underlying these protective effects.

### 4.4. Qualitative Analysis of Micro-CT Images

Three-dimensional micro-CT imaging provides a definitive spatial assessment of bone microarchitecture, offering structural insights that bone mineral density or calcium content alone cannot fully capture. In this study, the qualitative 3D visual reconstructions of the femoral metaphyseal region clearly illustrate the destructive impact of estrogen deficiency and the protective efficacy of high-dose *Artocarpus integer* leaf extract (AIE-H).

#### 4.4.1. Microarchitectural Deterioration in the OVX Group

The untreated OVX group exhibited profound architectural degradation characterized by a severe loss of trabecular elements. The dense, highly interconnected, and isotropic trabecular lattice observed in the Sham group was replaced by a sparse, fragmented, and perforated structure. Visually, this manifest as a classic “plate-to-rod” transition, where the structurally superior trabecular plates thinned out and broke down into isolated, rod-like structures. This visual thinning and fragmentation directly correlate with the sharp reductions in bone volume fraction (BV/TV), trabecular thickness (Tb.Th), and trabecular number (Tb.N) observed in the quantitative analysis.

#### 4.4.2. Structural Preservation by AIE-H Treatment

Conversely, intervention with AIE-H demonstrated a striking preservation of the trabecular architecture. The 3D images of the AIE-H group revealed a well-maintained structural network with noticeably thicker trabecular plates, fewer microstructural gaps, and vastly superior connectivity compared to the untreated OVX group. The structural appearance of the AIE-H treated bone closely mirrored the healthy architectural patterns seen in the Sham control. Furthermore, the visual reduction in empty spatial voids within the medullary canal stands in perfect alignment with the significantly decreased trabecular separation (Tb.Sp) quantified in Table 6.

#### 4.4.3. Mechanistic Implications

The ability of AIE-H to prevent the structural collapse of the trabecular network suggests that its constituent bioactive flavonoids effectively counteract high-turnover bone resorption at this dosage. Estrogen withdrawal typically accelerates osteoclastic perforations of trabecular plates; once a plate is perforated, the structural template is lost, and the bone cannot easily rebuild its original microarchitecture. By preserving the spatial continuity and thickness of these trabecular plates, AIE-H demonstrates potent anti-resorptive properties, structurally validating the downstream alterations observed in osteoclastogenesis markers (*Trap*, *Ctsk*, and *Nfatc1*).

### 4.5. RT-PCR Validation of Osteogenic and Osteoclastogenic Genes

To further investigate the molecular mechanisms underlying the anti-osteoporotic activity of *A. integer*, the expression of genes associated with osteoblast differentiation (*Runx2* and *Osx*) and osteoclastogenesis (*Trap*) were evaluated. These genes were selected because they represent key molecular targets predicted by the network pharmacology analysis and are closely associated with bone remodeling.

The downregulation of *Runx2* and *Osx* observed in OVX rats is consistent with impaired osteoblast differentiation under estrogen-deficient conditions. *Runx2* is widely recognized as the master transcription factor regulating osteoblast commitment and bone formation, while Osterix (*Osx*) acts downstream of *Runx2* and is essential for osteoblast maturation and matrix mineralization. Reduced expression of these genes indicates suppression of osteogenic activity and contributes to the deterioration of bone mass observed after ovariectomy.

Treatment with *A. integer* increased the expression of both *Runx2* and *Osx*, suggesting the restoration of osteoblast differentiation and bone-forming activity. These findings are in agreement with the increased femoral calcium content observed in the treated groups and support the hypothesis that the extract promotes osteogenesis. Furthermore, the predicted interactions of *A. integer* phytochemicals with *Akt1*, *Igf1r*, *Fgfr1*, *Gsk3b*, and *Ctnnb1* provide a plausible mechanistic basis for the activation of osteogenic signaling pathways.

In contrast, ovariectomy increased the expression of the osteoclast-related genes *Trap. Nfatc1* is considered a master regulator of osteoclast differentiation and controls the expression of several downstream osteoclast-specific genes, including *Trap*. Elevated expression of these markers indicates enhanced osteoclastogenesis and bone resorption following estrogen deficiency. Increased osteoclast activity is a hallmark of postmenopausal osteoporosis and contributes directly to bone loss and deterioration of skeletal structure.

Treatment with *A. integer* reduced the expression of *Trap*, indicating suppression of osteoclast differentiation and resorptive activity. These findings are consistent with the network pharmacology results, which identified several antiresorptive targets, including *Ptgs2*, *Nlrp3*, *Mmp9*, *Mapk14*, *Src*, and *Il6*, that are involved in inflammatory signaling and osteoclast activation. The ability of the extract to downregulate osteoclastogenic markers suggests that its anti-osteoporotic activity may involve inhibition of excessive bone resorption in addition to stimulation of bone formation.

Taken together, the RT-PCR findings provide experimental support for the network pharmacology predictions and demonstrate that *A. integer* exerts its effects through multiple complementary mechanisms. The simultaneous upregulation of osteogenic markers (*Runx2* and *Osx*), and downregulation of osteoclastogenic markers (*Trap*) suggest that the extract promotes a favorable balance between bone formation and bone resorption under estrogen-deficient conditions. These molecular changes, together with improvements in calcium content and metabolic biomarkers, support the potential of *A. integer* as a multi-target natural intervention for osteoporosis.

### 4.6. Study Limitations and Future Directions

Although the present study provides promising evidence supporting the anti-osteoporotic potential of *Artocarpus integer* leaf extract, several limitations should be considered. First, the phytochemical constituents identified by LC–HRMS were tentatively annotated based on accurate mass measurements, retention times, and database matching. While this approach provides valuable information regarding the chemical composition of the extract, structural confirmation using authentic standards and spectroscopic techniques such as NMR is still required. Therefore, the bioactive compounds identified in this study should be regarded as putative candidates until further validation is performed.

Another limitation relates to the predictive nature of the network pharmacology analysis. Although the compound–target network identified several biologically relevant pathways associated with osteoporosis, including estrogen signaling, osteoblast differentiation, osteoclastogenesis, inflammation, oxidative stress, and extracellular matrix remodeling, these interactions were inferred from existing databases and computational models. Consequently, the predicted mechanisms should be interpreted as hypotheses that require experimental confirmation. Nevertheless, the integration of network pharmacology with in vivo observations provides a useful framework for understanding the potential multi-target actions of *A. integer*.

The current work also represents an initial evaluation of the anti-osteoporotic effects of *A. integer* in ovariectomized rats. While body weight, uterine weight, and osteopontin measurements provide important information regarding estrogen-deficiency status and bone metabolism, these parameters alone cannot fully reflect changes in bone quality and structural integrity. Osteoporosis is characterized not only by alterations in biochemical markers but also by deterioration of bone mineral content and microarchitecture. Therefore, further assessment of femoral calcium content and tibial microCT parameters, including bone volume fraction (BV/TV), trabecular thickness (Tb.Th), trabecular separation (Tb.Sp), bone mineral density (BMD), and connectivity, is necessary to provide more direct evidence of skeletal preservation.

In addition, molecular validation remains essential for confirming the mechanisms predicted by network pharmacology. The planned RT-PCR analysis of *Runx2*, *Osx*, and *TRAP* will help clarify whether *A. integer* promotes osteoblast differentiation, suppresses and osteoclastogenesis signaling pathways. These molecular findings will provide important evidence linking the predicted targets to the observed biological effects.

The pharmacokinetic properties and bioavailability of the identified phytochemicals were also not investigated in the present study. This is particularly relevant because many flavonoids and polyphenolic compounds undergo extensive metabolism and may exhibit limited oral bioavailability. Future studies should therefore evaluate absorption, metabolism, tissue distribution, and active metabolites to better understand the relationship between phytochemical exposure and therapeutic efficacy.

Because osteoporosis is a chronic condition requiring long-term management, additional studies examining prolonged administration and safety are also warranted. Evaluation of hepatic, renal, metabolic, and reproductive parameters, together with histopathological assessment of bone and estrogen-responsive tissues, would provide important information regarding the long-term safety profile of the extract. Such investigations are especially important for identifying therapies that preserve bone without causing undesirable estrogenic effects in reproductive organs.

Future research should also focus on bioactivity-guided fractionation and isolation of the major active compounds responsible for the observed effects. Prenylated flavonoids and chalcone derivatives, particularly isobavachalcone, artocarpesin, and morachalcone A, emerged as promising candidates from both LC–HRMS and network pharmacology analyses. Further studies involving molecular docking, target-binding assays, osteoblast–osteoclast co-culture systems, and mechanistic gene-silencing approaches would help clarify their specific roles in bone remodeling.

Despite these limitations, the present study provides a valuable foundation for understanding the anti-osteoporotic potential of *A. integer*. By combining phytochemical profiling, network pharmacology, physiological evaluation, and planned molecular and structural analyses, this work highlights the potential of *A. integer* as a multi-target natural intervention for osteoporosis. The findings support further preclinical investigation and may contribute to the future development of plant-derived therapies for the prevention and management of postmenopausal osteoporosis.

## 5. Conclusions

**Figure 5:**
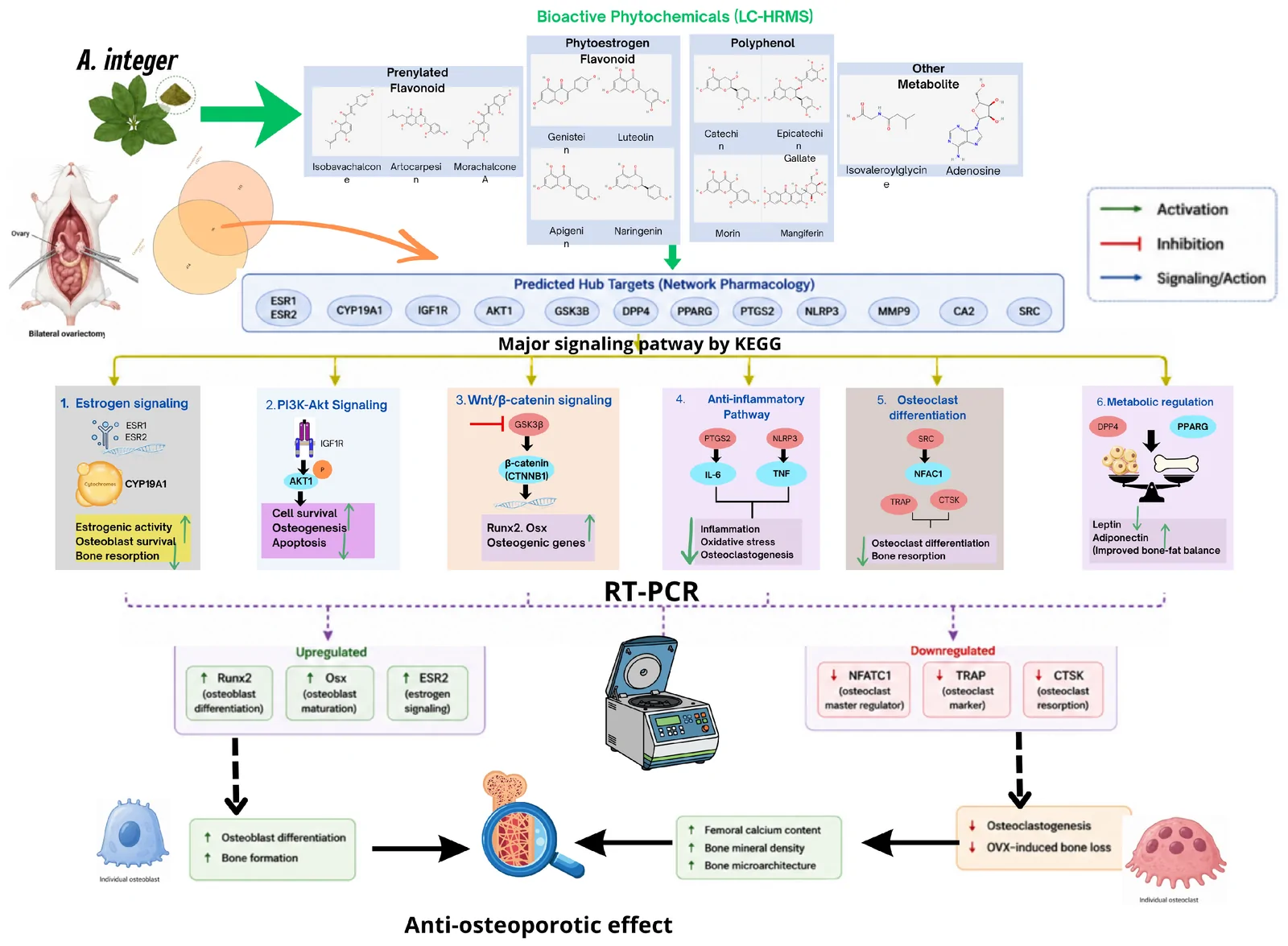
Proposed mechanism of action.

*Artocarpus integer* leaf extract exhibited promising anti-osteoporotic activity in ovariectomized rats. LC–HRMS and network pharmacology analyses suggested that the extract acts through multiple pathways related to estrogen signaling, osteoblast differentiation, osteoclastogenesis, inflammation, and oxidative stress. The proposed mechanism shown in Figure 5 belongs to prenylated chalcone and flavonoid classes (Table 2). Treatment improved metabolic parameters, preserved femoral calcium content, increased the expression of osteogenic genes (*Runx2* and *Osx*), and suppressed osteoclastogenic markers (*TRAP)*. Collectively, these findings indicate that *A. integer* may protect against estrogen-deficiency-induced bone loss through coordinated regulation of bone formation and bone resorption, supporting its potential as a natural candidate for osteoporosis management.

## Figures and Tables

**Table 1:** Primer sequences used for quantitative real-time PCR analysis.

Gene	Direction	Primer Sequence (5′–3′)
*Runx2*	Forward	CCTGAACTCAGCACCAAGTCCT
	Reverse	TCAGAGGTGGCAGTGTCATCA
*Osx*	Forward	CTGGGAAAAGGAGGCACAAAGA
	Reverse	GGGGAAAGGGTGGGTAGTCATT
*TRAP*	Forward	CACTCCCACCCTGAGATTTGT
	Reverse	CATCGTCTGCACGGTTCTG

Abbreviations: *Runx2*, runt-related transcription factor 2; *Osx*, osterix; *TRAP*, tartrate-resistant acid phosphatase.

**Table 2:** Tentatively identified phytochemical constituents of *Artocarpus integer* leaf extract with potential relevance to bone metabolism determined by LC–HRMS analysis.

No.	Compound Name	Molecular Formula	RT (min)	*m/z*	Peak Area	Compound Class	Reported Pharmacological Relevance to Bone
				[M + H]			
1	Isovalerylglycine	C_7_H_13_NO_3_	0.72	160.0965	5.15 × 10^9^	Amino acid derivative	Metabolic biomarker; possible indirect metabolic–bone association
2	Epicatechin	C_15_H_14_O_6_	0.78	291.0859	5.65 × 10^8^	Flavan-3-ol	Osteoblast support and antioxidant activity
3	Adenosine	C_10_H_13_N_5_O_4_	1.04	268.1037	2.47 × 10^7^	Nucleoside	Bone remodeling signaling and osteoblast regulation
4	Catechin	C_15_H_14_O_6_	1.18	289.0715	6.72 × 10^8^	Flavan-3-ol	Antioxidant protection against bone loss
5	Mangiferin	C_19_H_18_O_11_	2.94	421.0766	2.55 × 10^6^	Xanthone glycoside	Osteoprotective antioxidant and anti-inflammatory activity
6	Epigallocatechin gallate (EGCG)	C_22_H_18_O_11_	3.43	457.0766	2.35 × 10^6^	Polyphenolic catechin	Inhibits osteoclastogenesis and inflammatory bone resorption
7	Genistein	C_15_H_10_O_5_	5.41	269.0452	1.38 × 10^6^	Isoflavone	Phytoestrogen; stimulates osteoblast differentiation and suppresses osteoclast activity
8	Morin	C_15_H_10_O_7_	5.93	301.0351	2.41 × 10^6^	Flavonol	Antioxidant and osteoprotective activity
9	Luteolin	C_15_H_10_O_6_	5.97	285.0401	6.62 × 10^7^	Flavone	Suppresses osteoclast differentiation and oxidative stress
10	Morachalcone A	C_20_H_20_O_5_	6.4	341.1747	1.65 × 10^6^	Chalcone	Osteogenic and antioxidant potential
11	Apigenin	C_15_H_10_O_5_	6.73	269.0453	1.02 × 10^8^	Flavone	Inhibits osteoclastogenesis (NFATC1/*TRAP* signaling), anti-inflammatory activity
12	Naringenin	C_15_H_12_O_5_	6.84	271.0608	5.95 × 10^6^	Flavanone	Promotes osteoblast differentiation and bone mineralization
13	Artocarpesin	C_20_H_18_O_6_	9.13	355.154	1.46 × 10^7^	Prenylated flavonoid	Osteogenic and anti-inflammatory potential
14	Isobavachalcone	C_20_H_20_O_4_	10.48	325.1434	6.53 × 10^7^	Prenylated chalcone	Osteoblast differentiation, anti-osteoclastogenesis, anti-inflammatory activity

Abbreviations: RT, retention time; *m/z*, mass-to-charge ratio.

**Table 3:** Functional clustering of intersecting osteoporosis-related targets identified by MCL clustering analysis in Cytoscape.

Cluster	Representative Genes	Functional Annotation	Relevance to Osteoporosis
Cluster 1	*Nlrp3*, *Ikbkb*, *Casp1*, *Il2*, *Il5*, *Il6*, *Stat1*, *Ptgs2*, *Ctsb*, *Ctss*, *Mapk1*, *Mapk14*, *Nfe2l2*, *Gsk3b*, *Mmp9*, *Serpine1*, *Cxcl12*, *Cxcr4*, *Ctnnb1*, *Gli1*, *Casp3*, *Parp1*, *Dnmt1*, *Hif1a*, *Bcl2*, *Bcl2l1*	Osteoclastogenesis, inflammatory signaling, oxidative stress, and osteogenic regulation	Integrates inflammatory bone resorption (NF-κB/NLRP3/PTGS2), matrix remodeling, oxidative stress response, apoptosis regulation, and osteoblast signaling
Cluster 2	*Esr1*, *Esr2*, *Esrra*, *Nr1h4*, *Shbg*	Estrogen signaling and steroid hormone regulation	Regulates estrogen-deficiency-related bone remodeling and postmenopausal osteoporosis
Cluster 3	*Sirt3*, *Sirt6*, *Nampt*, *Nt5e*, *Enpp1*	Bone metabolism, mineralization, and metabolic homeostasis	Associated with osteoblast metabolism, oxidative protection, and bone mineral regulation
Cluster 4	*Fgfr1*, *Pik3ca*, *Pik3cd*, *Pik3cg*, *Ptk2*	PI3K–Akt signaling and osteoblast differentiation	Promotes osteoblast proliferation, survival, anabolic signaling, and skeletal development
Cluster 5	*Adamts4*, *Adamts5*, *Mmp3*, *Mmp13*	Extracellular matrix remodeling and bone degradation	Mediates collagen degradation and trabecular microarchitecture deterioration
Cluster 6	*Dpp4*, *Mgam*, *Slc5a2*	Metabolic–bone axis and glucose metabolism	Connects metabolic regulation with bone remodeling and osteoporosis risk
Cluster 7	*Ppara*, *Ppard*	Lipid metabolism and bone–fat regulation	Regulates osteoblast–adipocyte balance and metabolic skeletal homeostasis
Cluster 8	*Prkaca*, *Ptger4*	Hormonal signaling and bone remodeling	Regulates cyclic AMP signaling and prostaglandin-mediated bone metabolism
Cluster 9	*Hsd17b2*, *Cyp19a1*	Estrogen biosynthesis and steroid metabolism	Modulates estrogen availability and estrogen-deficiency-related bone loss

**Table 4:** Top enriched biological processes and signaling pathways associated with osteoporosis-related targets.

Category	Pathway	Gene Count	FDR/*p*-Value	Related Genes
KEGG	Osteoclast differentiation	12	<0.001	*Src*, *Ptgs2*, *Mapk1*
KEGG	Estrogen signaling pathway	10	<0.001	*Esr1*, *Esr2*, *Cyp19a1*
KEGG	PI3K-Akt signaling	15	<0.001	*Igf1r*, *Akt1*
GO-BP	Bone remodeling	11	<0.001	*Mmp2*, *Mmp9*
GO-BP	Response to oxidative stress	9	<0.01	*Nfe2l2*

**Table 5:** Effect of *Artocarpus integer* leaf extract on femoral calcium content in ovariectomized rats following 28 days of daily oral treatment.

Group	Calcium Content (%)	Recovery vs. Sham (%)
Sham	59.77 ± 8.02	100
OVX	43.02 ± 5.42 *	71.9
Raloxifene	51.19 ± 4.41	85.6
AIE-L	53.32 ± 3.25 #	89.2
AIE-M	63.11 ± 12.88 #	105.6
AIE-H	52.73 ± 2.70 #	88.2

Note: Data are expressed as mean ± SD (n = 6). * p<0.05 vs. SHAM group; # p<0.05 vs. Negative Control group using one-way ANOVA followed by Tukey’s post hoc test.

**Table 6:** Quantitative micro-CT bone microarchitecture parameters of the proximal tibia across experimental groups following 28 days of daily oral treatment.

	BV/TV (%)	Tb.Th (mm)	Tb.N (1/mm)	Tb.Sp (mm)	Total Porosity (%)
SHAM	40.54 ± 2.91	0.23 ± 0.005	2.02 ± 0.008	0.53 ± 0.09	65.35 ± 0.53
OVX	34.65 ± 1.94 *	0.19 ± 0.01 *	1.62 ± 0.06 *	0.34 ± 0.06 *	59.46 ± 0.07 *
Raloxifene	39.64 ± 1.72 #	0.22 ± 0.02 #	1.78 ± 0.06 #	0.51 ± 0.05 #	60.36 ± 0.31 #
AIE-H	41.11 ± 3.27 #	0.25 ± 0.02 #	1.69 ± 0.27	0.47 ± 0.09 #	58.89 ± 0.44

Note: BV/TV, Bone Volume/Total Volume; Tb.Th, Trabecular Thickness; Tb.N, Trabecular Number; Tb.Sp, Trabecular Separation. * p<0.05 vs. SHAM group;  p #<0.05 vs. Negative Control group.

**Table 7:** Effect of *Artocarpus integer* leaf extract (AIE) on relative bone-related mRNA expression levels in ovariectomized rats.

Group	*Runx2*	*Osx*	*Trap*
SHAM	1.03 ± 0.30	1.05 ± 0.23	1.02 ± 0.18
OVX	0.52 ± 0.76	0.85 ± 0.08	1.67 ± 1.06
RAL	0.86 ± 0.67	1.51 ± 0.18	1.08 ± 0.43
AIE-H	0.92 ± 0.49	1.10 ± 0.19	1.53 ± 0.43

Note: Data represent the relative fold-change in mRNA expression levels normalized against *Actb* via the $2^{-\Delta \Delta C_{T}}$ method. Values are expressed as mean ± standard deviation (SD).

## Data Availability

All data generated or analyzed during this study are included in this published article and its Supplementary Materials.

## Supplementary Materials

The following supporting information can be downloaded at: https://www.mdpi.com/article/10.3390/nu18172874/s1.
